# Human Keratinocyte Growth and Differentiation on Acellular Porcine Dermal Matrix in relation to Wound Healing Potential

**DOI:** 10.1100/2012/727352

**Published:** 2012-05-03

**Authors:** Robert Zajicek, Vaclav Mandys, Ondrej Mestak, Jan Sevcik, Radana Königova, Eva Matouskova

**Affiliations:** ^1^Prague Burn Centre, 3rd Faculty of Medicine, Charles University in Prague, 100 00 Prague 10, Czech Republic; ^2^Prague Burn Centre, University Hospital Kralovske Vinohrady, 100 00 Prague 10, Czech Republic; ^3^Department of Pathology, 3rd Faculty of Medicine, Charles University in Prague, 100 00 Prague 10, Czech Republic; ^4^Department of Plastic Surgery, 1st Faculty of Medicine, Charles University in Prague, 180 00 Prague 2, Czech Republic

## Abstract

A number of implantable biomaterials derived from animal tissues are now used in modern surgery. Xe-Derma is a dry, sterile, acellular porcine dermis. It has a remarkable healing effect on burns and other wounds. Our hypothesis was that the natural biological structure of Xe-Derma plays an important role in keratinocyte proliferation and formation of epidermal architecture *in vitro* as well as *in vivo*. The bioactivity of Xe-Derma was studied by a cell culture assay. We analyzed growth and differentiation of human keratinocytes cultured *in vitro* on Xe-Derma, and we compared the results with formation of neoepidermis in the deep dermal wounds treated with Xe-Derma. Keratinocytes cultured on Xe-Derma submerged in the culture medium achieved confluence in 7–10 days. After lifting the cultures to the air-liquid interface, the keratinocytes were stratified and differentiated within one week, forming an epidermis with basal, spinous, granular, and stratum corneum layers. Immunohistochemical detection of high-molecular weight cytokeratins (HMW CKs), CD29, p63, and involucrin confirmed the similarity of organization and differentiation of the cultured epidermal cells to the normal epidermis. The results suggest that the firm natural structure of Xe-Derma stimulates proliferation and differentiation of human primary keratinocytes and by this way improves wound healing.

## 1. Introduction

A number of implantable biomaterials derived from human and animal tissues—such as acellular matrices derived from the human dermis (Alloderm), porcine dermis (Strattice, Permacol), porcine small intestine submucosa (Surgisis), or bovine pericardium (Veritas)—are used in modern surgery. These biomaterials are reasonably biocompatible and stable enough for long-term tissue replacements. They are used, for instance, in the field of reconstructive surgery for hernia repair [1, 2], soft tissue augmentation, breast reconstruction [3], and wound treatment [4, 5]. These biomaterials consist primarily of collagen structures and glycoproteins that maintain the structure of the original intercellular tissue to a greater or lesser extent.

Recently, a new type of acellular biomaterial was developed in the Czech Republic under the name of Xe-Derma (manufactured by MEDICEM Institute Ltd.). This anisotropic acellular biomaterial is a result of the research and development of recombinant skin using xenodermis and cultured keratinocytes at the Prague Burn Centre [6–9]. However, the regulations controlling the use of cultured cells in clinical practice are becoming increasingly complex. Our goal, then, was to develop an acellular dermis that would maximally support the growth of patient's own keratinocytes from the adnexa remnants in the wound by providing optimal conditions for their attachment, proliferation, and migration.

**Figure 1:**
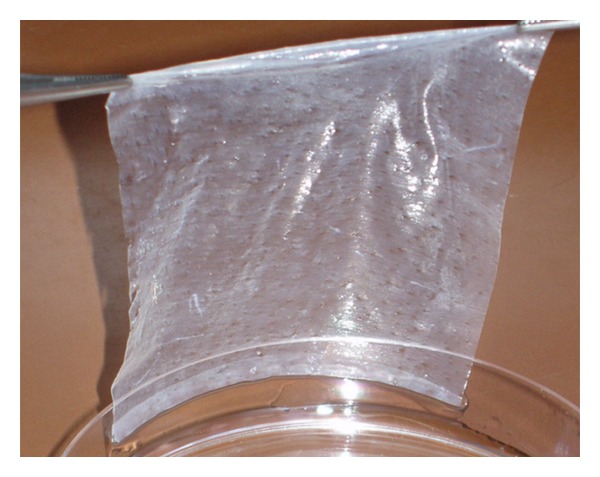
Acellular porcine dermis Xe-Derma. Hydrated XD has properties similar to those of normal human skin.

**Figure 2:**
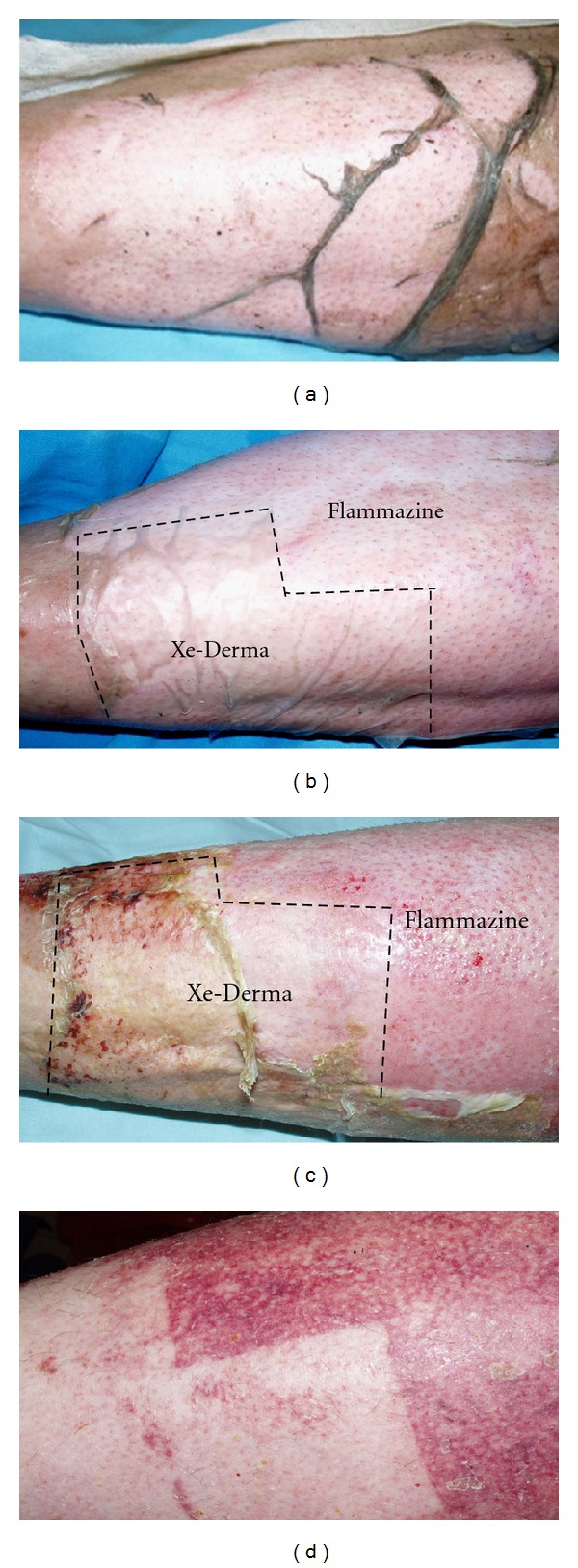
The effect of XD compared to antibacterial cream Flammazine in a patient burned by acetone steam (deep dermal burn). An example of XD healing effect. (a) Day 1: the burn wound on the leg at the initiation of treatment. (b) XD was applied to the distal part of the leg, and the proximal part was covered with silver sulphadiazine (Flammazine). (c) Day 9: the area under XD healed, while the area under Flammazine did not heal until day 18. The XD dressing was not changed during the treatment, while the Flammazine dressing was changed every second day. (d) Day 20: detail of the healed area. The esthetic result and the speed of healing under XD are notable.

Xe-Derma (XD) is a dry, sterile, commercially available acellular porcine dermis (Figure 1). Hydrated XD displays biomechanical properties similar to those of human skin. In the clinical study, we have shown that XD has significantly higher efficacy in partial thickness burns in children than hydrocolloid dressing (larger area is healed in comparable time), and no exchange of the cover is needed until complete healing [10]. XD spontaneously adheres to the wound and immediately prevents bleeding. After the wound becomes epithelized, the dry xenodermis peels off, usually within 4–14 days [10, 11]. In comparison with the silver sulphadiazine cream Flammazine (Solvay Pharmaceuticals B.V., The Netherlands), the gold standard for the treatment of deep dermal burns, XD induces rapid healing with superior cosmetic results (Figure 2). Since 2007, we have successfully used XD in the treatment of burns [10], leg ulcers, diabetic foots, dermabrasions and donor sites for skin grafts [11].

The aim of the current study was to gain more insight into the biological mechanism of XD-mediated wound healing. The bioactivity of XD has been demonstrated in a cell culture assay. Keratinocyte growth and differentiation *in vitro* on XD and *in vivo *under XD was analyzed by histology and immunocytochemistry. Our hypothesis was that the natural biological structure of the dermis plays an important role in proliferation and differentiation of the patient's own keratinocytes.

## 2. Material and Methods

We analyzed growth and differentiation of primary human keratinocytes cultured *in vitro* on XD. We then compared the results with differentiation of neoepidermis in a freshly healing wound treated with XD. Proliferation and differentiation of skin cells *in vitro* and in the deep dermal burn wound covered with XD was compared using routine histological and immunohistological methods.

### 2.1. Tensile Strength of XD

Tensile testing on bone-shaped samples was conducted on the testing engine INSPEKT desk 10 kN (Hegewald & Peschke) equipped with programme Labmaster. Samples were hydrated overnight in PBS and pulled to failure at 100 mm/min using a mechanical stand with a 100 N load cell. In total, 40 samples of XD were measured.

### 2.2. Keratinocyte Cultivation on XD

Human primary keratinocytes (2nd passage) were obtained from redundant skin of donors undergoing abdominoplasty. Keratinocytes were cultured on XD using the 3T3 feeder layer technique [6, 12]. Prior to seeding the cells, XD in the tissue culture dish was immersed in a standard culture medium (HMEM with non-essential amino acids, 0.12 g/L sodium pyruvate, 1 g/L NaHCO_3_, and 10% bovine serum) overnight at 37°C. Lethally irradiated NIH-3T3 cells were used as feeder cells and were seeded on the dish with XD at a concentration of 3 × 10^4^ cells/cm^2^ in a standard culture medium. On the following day, the keratinocytes were seeded at a concentration of 5 × 10^4^ cells/cm^2^ in a standard culture medium enriched with 2% fetal bovine serum, hydrocortisone (0.5 ug/mL), insulin (0.12 U/mL), cholera toxin (10^−10^ M), and EGF (5 ng/mL). The cultures were grown in a humidified 3.5% CO_2_ atmosphere. The progress of cell growth on XD was followed on the noncovered areas of the dish or on specimens stained by May-Grünwald and Giemsa-Romanowski.

After achieving confluence, XD, along with 1-2 keratinocyte layers, was cut into pieces of approximately 1 cm^2^ and lifted to the air-liquid interface on a stainless steel grid covered with two layers of sterile gauze. The “skin” was cultured at the air-liquid interface for one week.

### 2.3. Keratinocyte Cultivation on Biopad

In an *in vitro* control sample, keratinocytes were cultured on the equine collagen Biopad, a sponge-shaped lyophilized equine collagen type I, using the same methodology as the keratinocyte cultivation on XD.

### 2.4. Treatment of Burns with XD

In deep dermal burns (classified as mixed burns of degrees 2b and 3), the necrotic tissue was surgically removed, and XD was used to cover the area after necrectomy to prepare the wound for skin grafting. XD was hydrated for 1–3 min in saline and applied to the wound. The dressing was covered with one layer of tulle gras and with plain gauze wetted with 3% boric acid. As a control, part of the wound was covered by tulle gras Grassolind and plain gauze with 3% boric acid only. Biopsies of three patients were taken in the course of one week after XD application.

### 2.5. Histology

Specimens of XD with *in vitro* cultured keratinocytes and samples from three deep dermal wounds after necrectomy covered with XD (without cultured keratinocytes) or Grassolind were fixed in 10% buffered formaldehyde and processed by the routine histological technique. Five-micron-thick paraffin sections were mounted on glass histological slides and stained with hematoxylin and eosin, using the van Gieson/orcein method or used for immunohistochemical staining.

### 2.6. Immunohistochemical Staining

The standard immunohistochemical technique was performed using antibodies for the detection of high-molecular-weight cytokeratins (HMW CKs, clone 34*β*E12, Dako, Denmark), nuclear antigen p63 (Ab-1, clone 4A4, NeoMarkers, Fremont, CA, USA), CD29 (Novocastra, Newcastle upon Tyne, UK), and involucrin (Novocastra, UK). N-Histofine immunohistochemical staining reagent (Nichirei Biosciences, Tokyo, Japan) and 3-3′diaminobenzidine as a chromogen were used to visualize the immunohistological reaction.

## 3. Results

### 3.1. Biomechanical Properties and Structure of XD

**Figure 3:**
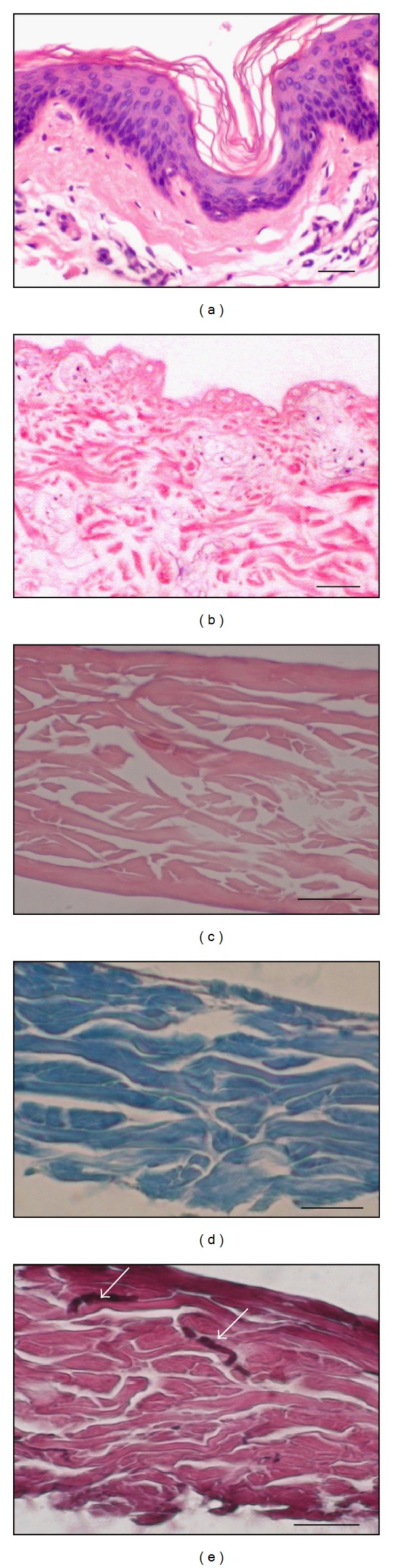
Structure of XD. Histological sections of (a) porcine skin, (b) acellular xenodermis immediately after removal of epidermis and other cells, (c) XD (hematoxylin and eosin), (d) XD stained with trichrome shows the majority of collagen fibres (blue), (e) XD stained with van Gieson/orcein stain shows fragments of elastic fibres (arrows). Bar—30 *μ*m.

XD was prepared from xenografts as dry acellular porcine dermis (Figure 1). After rehydration, the biomechanical features of XD (elasticity, adherence, and haemostatic effect) resemble those of normal human skin. Thickness of hydrated XD was 0.25–0.35 mm. Tensile strength was between 6.6 ± 1.2 MPa. Histological slides stained with hematoxylin and eosin and by van Gieson/orcein method showed that XD is a 3D matrix formed of a natural biological network of collagen fibres and fragments of elastic fibres (Figure 3).

### 3.2. Histological Examinations *In Vivo* and *In Vitro*

**Figure 4:**
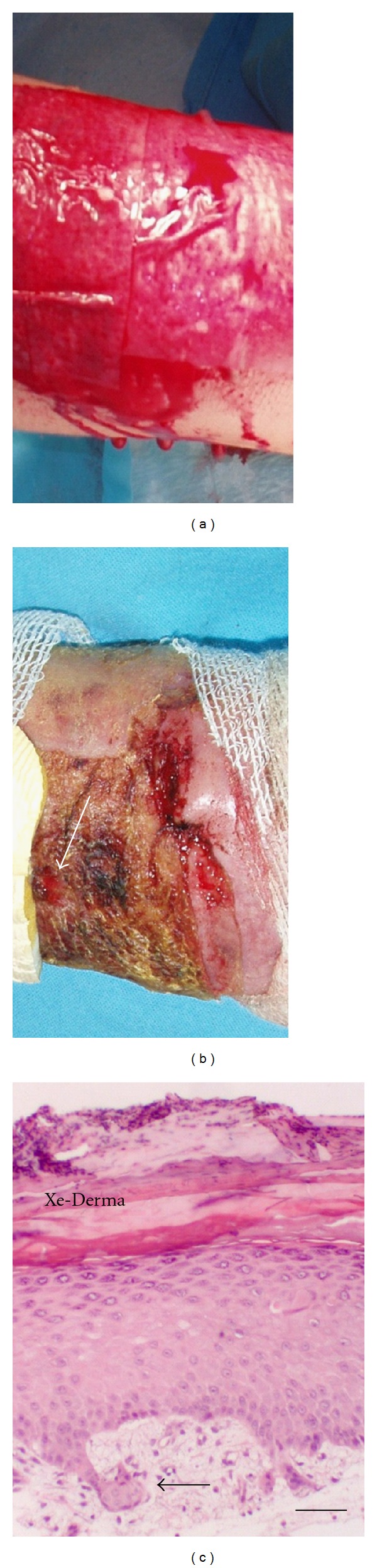
Xe-Derma used to cover areas after necrectomy to prepare the wound for autografting. (a) Temporary cover of an area after necrectomy originally evaluated as 3rd degree burn. XD was adhered immediately and stopped bleeding instantly. (b) XD incrusted by fibrin absorption (arrow—the point of biopsy on day 8). (c) Histology taken on day 8 showed fully differentiated epidermis without or with low development of rete ridges (arrow). Bar—50 *μ*m.

*In vivo: *in wounds treated with XD, histological studies one week after application revealed neoepidermis without or with low development of rete ridges. XD remained attached to the wound (Figure 4). In wounds treated with Grassolind, no epidermis was formed. 

**Figure 5:**
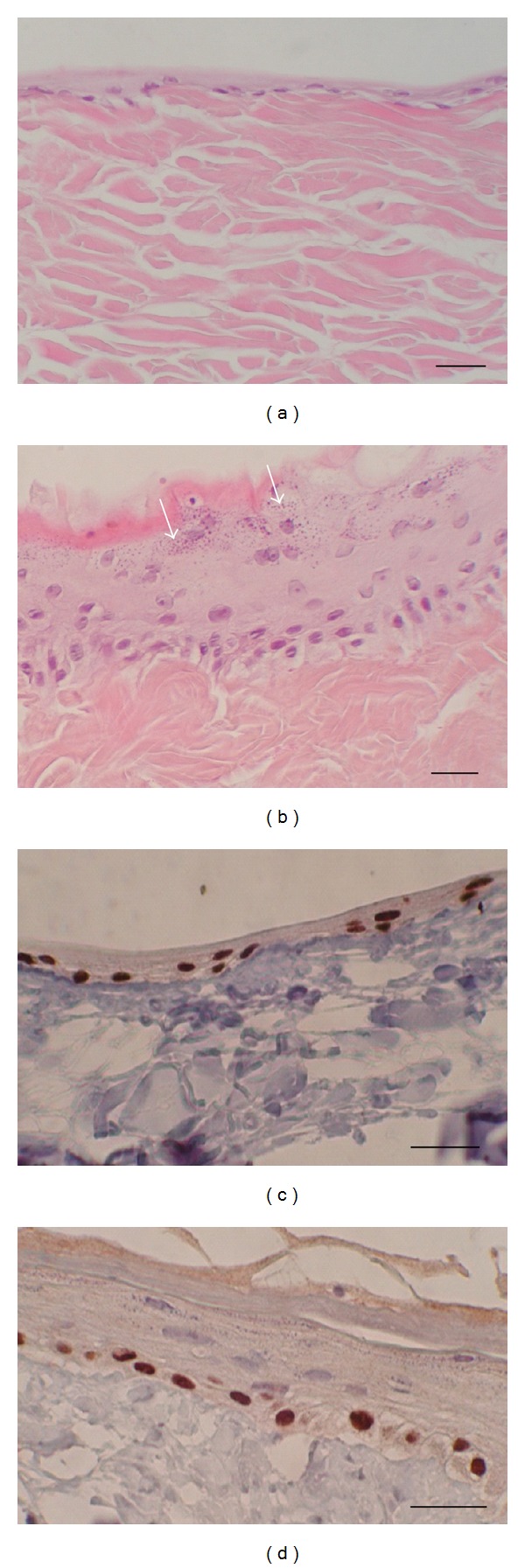
Keratinocytes cultured and differentiated on XD. (a) Keratinocytes grown submerged formed 1-2 layers on XD. (b) Keratinocytes grown at the air-liquid interface formed the basal, spinous, and granular layers. Keratohyalin granules (arrows) in the granular layer are notable. (c) Keratinocytes grown submerged stained immunocytochemically for p63 (nuclear protein p63 is expressed in basal cells). (d) Keratinocytes grown at the air-liquid interface stained for p63. Bars—30 *μ*m.

*In vitro: *keratinocytes that were grown submerged in the medium formed 1-2 cell layers on the XD matrix (Figures 5(a) and 5(c)). After the cultures were lifted to the air-liquid interface, the epidermis became stratified and formed 5–15 cell layers in the course of one week (Figures 5(b) and 5(d)). The multilayered structure closely resembled well-differentiated epidermis *in vivo* composed of distinct basal, spinous, granular, and cornified layers (Figure 5).

### 3.3. Expression and Distribution of Keratinocyte Differentiation Markers

**Figure 6:**
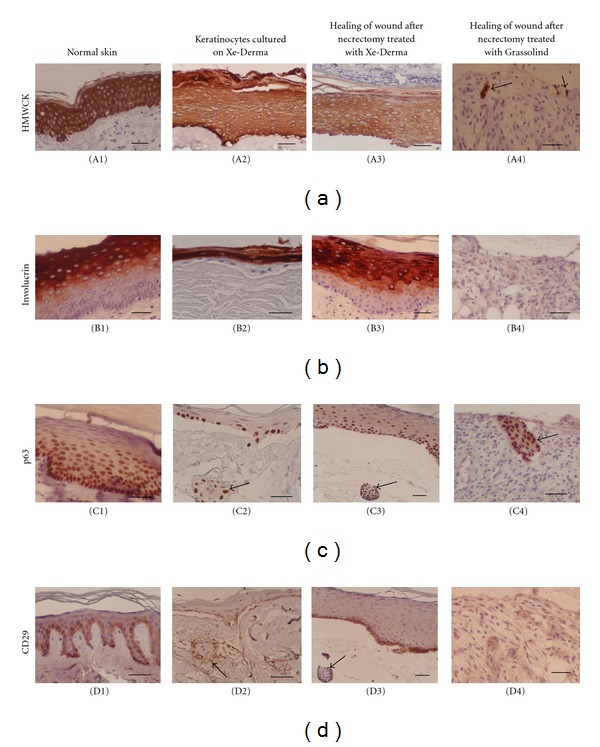
Expression of differentiation markers of the keratinocytes cultured on XD at the air-liquid interface (A2, B2, C2, D2) compared to the normal skin (A1, B1, C1, D1), to freshly healing wound after necrectomy treated with XD (A3, B3, C3, D3), and to freshly healing wound after necrectomy treated with Grassolind (A4, B4, C4, D4). The wounds after necrectomy were covered with XD (column 3) or tulle gras Grassolind (column 4). On day 8 after treatment, biopsies were taken. The expression of differentiation markers HMW CK (a), involucrin (b), p63 (c), and CD29 (d) was compared in the normal skin (column 1), in keratinocytes cultured on XD (column 2), in the wound treated with XD (column 3), and in the wound treated with Grassolind as a control (column 4). The figures show that keratinocyte growth, and differentiation *in vitro* on XD (column 2) and *in vivo* epithelization under XD (column 3) is similar and that the wound covered with XD formed epidermis within one week (column 3), while the wound covered with Grassolind did not epithelize (column 4), although remnants of keratinocytes (A4) or adnexa remnants (C4) are visible in the dermis (arrows). Note that cells migrated into the XD matrix formed islands resembling primitive gland-like structures (C2, D2—arrows); they are p63− in the centre and p63+ at the outer margin of the colony (compare to gland remnants in C3 and D3—arrows). Bars—50 *μ*m.

Paraffin sections of the normal skin, recombined skin (keratinocytes cultured on XD at the air-liquid interface), and of the wounds treated with XD or Grassolind were analyzed using a panel of antibodies detecting selected markers of keratinocyte differentiation. The reactivity of the antibodies in the cultured keratinocytes and in the freshly healing skin is shown in Table 1. Immunohistochemical staining of HMW CK, involucrin, p63, and CD29 of keratinocytes cultured on XD confirmed the organization and differentiation of keratinocytes similar to that of normal epidermis (Figure 6). HMW CK was strongly expressed in all keratinocytes cultured on XD (Figure 6(a)/(A2)). Involucrin was expressed in the granular and horny layers (Figure 6(b)/(B2)). CD29 was positive in the basal layer of keratinocytes (Figure 6(d)/(D2)). A similar differentiation pattern was observed in the newly formed epidermis after treatment with XD (Figure 6(a)/(A3), Figure 6(b)/(B3), Figure 6(d)/(D3)). The wound treated with Grassolind did not epithelize, although adnexa remnants or individual keratinocytes were identified in the biopsies (Figure 6(a)/(A4), Figure 6(c)/(C4)).

The nuclear protein p63 was expressed *in vitro* in all keratinocytes of the submerged cultures and in the basal layer of the cultures grown at the air-liquid interface (Figure 6(c)/(C2)). *In vivo*, p63 was expressed not only in the basal layer but also in a subpopulation of suprabasal cells (Figure 6(c)/(C3)). Keratinocytes *in vitro* occasionally migrated into the XD matrix and formed solid nests or islands (Figure 6(c)/(C2), Figure 6(d)/(D2)) resembling gland-like structures (Figure 6(c)/(C3), Figure 6(d)/(D3)).

### 3.4. Keratinocyte Growth on Biopad

**Figure 7:**
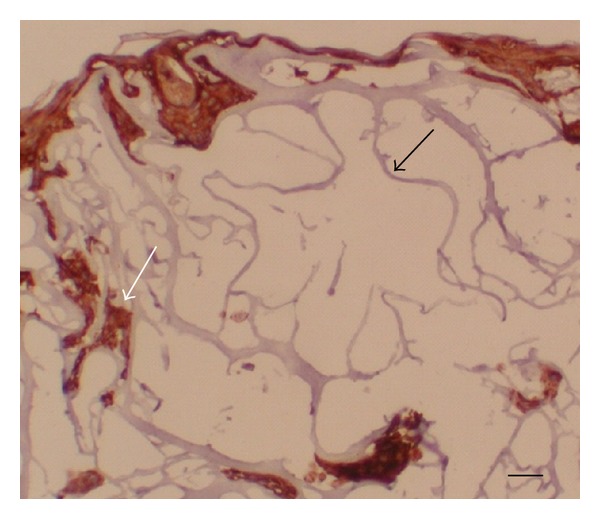
Keratinocytes cultured on Biopad (equine collagen). Note the fragile collagen fibers (black arrow) and the chaotic distribution of the irregular groups of keratinocytes. Immunohistological detection of involucrin shows terminally differentiated keratinocytes in the inner structure of Biopad (white arrow). Bar—30 *μ*m.

In the control sample, human keratinocytes were simultaneously cultured on Biopad. After two weeks of cultivation (one week submerged and one week at the air-liquid interface), the cells showed chaotic distribution. They migrated into the Biopad structure composed of fragile collagen fibers. Within the inner structure of this collagen, the cells were terminally differentiated, as shown by staining for involucrin (Figure 7).

## 4. Discussion

Xenografts have been used in the Prague Burn Centre since 1973 [13] for treatment of burns and other acute and chronic skin defects [11]. After 32 years of extensive use, the utilization of porcine xenografts was discontinued due to the regulations of the European Union. Since then, various synthetic materials have been used as temporary covers, but they are not comparable to xenografts because they do not display a similar level of biological activity.

Cell-free pig dermis was initially developed in our laboratory as a substrate for the cultivation of human keratinocytes [6]. The previously constructed recombined human/pig skin was used as a delivery system for the keratinocytes applied to accelerate the healing process [7–9]. However, dried xenodermis itself without keratinocytes has shown excellent healing properties.

The bioactivity of decellularized dermis materials can be demonstrated by a cell culture assay [14]. More detailed information on the cell behavior should contribute to the elucidation of the healing mechanism of the skin substitute. Our study has shown that XD, similarly as its laboratory predecessor cell-free pig dermis [6], is an appropriate substrate for keratinocyte attachment, growth, and differentiation. Morphological differentiation of keratinocytes has been achieved in organotypic culture systems grown at the air-liquid interface on various dermal-like substrates [15–17]. The cultured human keratinocyte/Xe-Derma organotypic skin model was studied using the criteria of tissue morphology and the presence and distribution of selected keratinocyte differentiation markers. The results were compared to the healing of deep dermal wounds after necrectomy covered with the XD dressing. The morphology of the keratinocyte-derived epidermis cultured *in vitro* at the air-liquid interface was very similar to the neoepidermis formed *in vivo* in the deep dermal burn from the remnants of keratinocytes in the residual skin adnexa. The expression and distribution of the markers HMW CK, CD29, and involucrin in keratinocytes cultured *in vitro *at the air-liquid interface corresponded to those expressed in the epidermis *in vivo*. The p63 nuclear antigen was expressed in the *in vitro* cultured epidermis in the basal layer only. In the neoepidermis developed *in vivo*, the p63 nuclear antigen was expressed in basal cells and in a fraction of suprabasal cells. Protein p63 is a marker of progenitor and transit-amplifying cells that are essential for the proliferative potential in the stratified epithelia [18–20]. This protein plays an important role in keratinocyte differentiation [20].

Cells usually do not grow on skin covers. Exceptions are biological covers including collagens; however, the attachment and cell proliferation depend on the particular structure, purity, type of storing, type of sterilization, and so forth. Differentiation of keratinocytes takes place only on materials similar to the normal dermis, usually in the presence of mesenchymal cells. We have tested several types of synthetic wound covers (e.g., Grassolind, Viacell, AskinaThinSite, Inadine, etc.) with negative results for cell growth. Limited cell growth was achieved on adhesive dressing Suprathel. Biopad was selected as comparative material for keratinocyte cultivation as it represents sponge-shaped (3D structured) pure collagen.

The fact that keratinocytes cultured on XD organized themselves in a manner similar to that of normal skin, while those cultured on the equine collagen Biopad grew in a disorderly fashion, supports our assumption that the natural biological structure of the dermis plays an important role in formation of the epidermal architecture. Signals from the underlying mesoderm seem to be required to trigger the discrete functions of p63 and other proteins in the ectoderm that play a key role in keratinocyte commitment, differentiation, and regeneration [20].

Our results are in accordance with the results of Feng et al. [21] documenting that porcine acellular dermal matrix used to cover deep second-degree burns preserves residual dermal tissue and epithelium and helps to accelerate the regeneration of epithelial and stem cells, thus shortening the healing time, remodeling skin structure, and consequently preventing hypertrophic scars at inception. One shot therapy without dressing exchange avoids secondary injury to the wound [21].

## 5. Conclusion

The firm natural structure of XD stimulates proliferation and differentiation of human primary keratinocytes. The keratinocytes cultured on XD organized themselves in a manner similar to that of normal skin, while those cultured on the equine collagen Biopad grew in a disorderly fashion. These results support our assumption that the natural biological structure of the dermis plays an important role in wound epithelization and formation of the epidermal architecture. The healing effects of XD result from the support of proliferation, migration, and differentiation of the patient's keratinocytes.

## Figures and Tables

**Table 1:** Keratinocyte differentiation* in vitro* and *in vivo*.

Antibody to	Reaction with keratinocytes on XD	Figure no.	Reaction with freshly healing skin epithelia	Figure no.
HMW CK	All keratinocytes	Figure 6(a)/(A2)	All keratinocytes	Figure 6(a)/(A3)
Involucrin	Granular and horny layer	Figure 6(b)/(B2)	Granular and horny layer	Figure 6(b)/(B3)
p63	Basal keratinocyts and islands inside the XD matrix	Figure 6(c)/(C2)	Basal and suprabasal keratinocytes, adnexa remnants	Figure 6(c)/(C3)
CD29	Basal cells and islands inside the XD matrix	Figure 6(d)/(D2)	Basal cells, adnexa remnants	Figure 6(d)/(D3)

Immunocytochemical markers of keratinocyte differentiation of keratinocytes cultured on XD eight days after lifting to the air-medium interface and in the freshly healing deep dermal wound eight days after treatment with XD.

## Abbreviations

XD: Xe-Derma
HMEM: Minimum essential medium in Hanks' salts
HMW CK: High-molecular-weight cytokeratins.
